# Involvement of DKK1 secreted from adipose‐derived stem cells in alopecia areata

**DOI:** 10.1111/cpr.13562

**Published:** 2023-11-22

**Authors:** Nahyun Choi, Juyeong Hwang, Doo Yeong Kim, Jino Kim, Seung Yong Song, Jong‐Hyuk Sung

**Affiliations:** ^1^ Epi Biotech Co., Ltd. Incheon South Korea; ^2^ College of Pharmacy, Yonsei Institute of Pharmaceutical Sciences Yonsei University Incheon South Korea; ^3^ New Hair Plastic Surgery Clinic Seoul South Korea; ^4^ Institute for Human Tissue Restoration, Department of Plastic and Reconstructive Surgery Yonsei University College of Medicine Seoul South Korea

## Abstract

Adipose‐derived stem cells (ASCs) have shown efficacy in promoting hair growth, while DKK1 inhibits the WNT pathway, which is associated with hair loss. Our study focused on investigating the expression of DKK1 in alopecia areata (AA), a condition characterised by significant increases in the DKK1 levels in human and mouse ASCs. Treatment of interferon‐γ increased the expression of DKK1 via STAT3 phosphorylation in ASCs. Treatment with recombinant DKK1 resulted in a decrease of cell growth in outer root sheath cells, whereas the use of a DKK1 neutralising antibody promoted hair growth. These results indicate that ASCs secrete DKK1, playing a crucial role in the progression and development of AA. Consequently, we generated DKK1 knockout (KO) ASCs using the Crispr/Cas9 system and evaluated their hair growth‐promoting effects in an AA model. The DKK1 KO in ASCs led to enhanced cell motility and reduced cellular senescence by activating the WNT signalling pathway, while it reduced the expression of inflammatory cytokines by inactivating the NF‐kB pathway. As expected, the intravenous injection of DKK1‐KO‐ASCs in AA mice, and the treatment with a conditioned medium derived from DKK1‐KO‐ASCs in hair organ culture proved to be more effective compared with the use of naïve ASCs and their conditioned medium. Overall, these findings suggest that DKK1 represents a novel therapeutic target for treating AA, and cell therapy using DKK1‐KO‐ASCs demonstrates greater efficiency.

## Graphical abstract

IFNγ stimulates an increase in DKK1 levels in adipose‐derived stem cells (ASCs) through the activation of the STAT3 pathway. The secreted DKK1 promotes inflammation and inhibits follicular growth. However, when DKK1 is knocked out in ASCs, it deactivates the NF‐kB pathway, resulting in reduced cytokine levels and suppression of the inflammatory response. Moreover, DKK1 knockout ASCs (DKK1‐KO‐ASCs) activate the Wnt pathway, enhancing the growth and migration abilities of the DKK1‐KO‐ASCs and reducing cellular senescence. As a result, DKK1‐KO‐ASCs exhibit an inhibitory effect on alopecia areata.

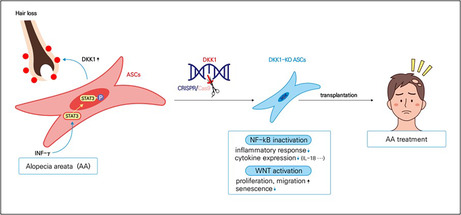

## 1 INTRODUCTION

Numerous studies have indicated the potential of adipose‐derived stem cells (ASCs) as a beneficial treatment for hair loss, with the ability to stimulate hair growth.^1^ ASCs have been found to promote the proliferation and migration of hair follicle cells, extend the anagen (growth) phase of the hair cycle and induce the differentiation of hair follicle stem cells.^1^, ^2^ Furthermore, ASCs improve the blood supply to hair follicles and alleviate inflammation, which are important factors for hair growth.^3^, ^4^ Additionally, ASCs secrete growth factors and cytokines that stimulate hair follicle growth and help hair loss prevention.^5^, ^6^, ^7^, ^8^, ^9^, ^10^, ^11^ Despite these findings, the effectiveness of ASCs in hair growth has been somewhat limited. To enhance the hair growth potential of ASCs, researchers have explored the use of various priming factors, including growth factors, cytokines and small molecules.^5^, ^6^, ^7^, ^8^, ^9^, ^10^, ^11^ Additionally, we conducted gene transfection experiments, introducing three genes (PDGF‐A, Sox2 and β‐catenin), to facilitate the differentiation of ASCs into dermal papilla cells (DPCs) and enhance the hair growth capabilities of ASCs.^12^

ASCs possess significant immunomodulatory properties, making them potential candidates for treating inflammatory or autoimmune diseases such as arthritis, colitis and other autoimmune disorders. In the context of alopecia areata (AA), ASCs and the stromal vascular fraction (SVF) of adipose tissue have been utilised to treat patients with this condition. Studies have reported positive outcomes in AA patients, including hair regeneration, increased hair growth and reduced pull test results, observed at 3 and 6 months after SVF treatment.^13^ Notably, a significant increase in hair diameter was observed specifically in women, and this effect was prominent 6 months post‐treatment. Additionally, Lee et al. demonstrated the effectiveness of conditioned medium from ASCs when combined with carbon dioxide fractional laser or microneedling, suggesting it as a potentially safe treatment option for refractory patches of AA.^14^ While there have been promising findings regarding the potential of ASCs in treating AA, their clinical application has not yet achieved satisfactory results. Therefore, further research and development are necessary to optimise the use of ASCs and improve the outcomes for AA patients.

DKK1 functions as a Wnt pathway antagonist by binding to the LRP5/6 co‐receptor, thereby inhibiting the interaction between the Wnt ligand and the Frizzled receptor, which leads to the suppression of Wnt signalling and prevents the stabilisation of β‐catenin, ultimately hampering its transcriptional activity.^15^ Recent studies have shed light on the crucial involvement of DKK1 in the regulation of hair growth. For instance, DKK1 hinders hair follicle enlargement and restricts the proliferation and abnormal positioning of hair follicle stem cells.^16^ Moreover, the administration of DKK1 during the early anagen phase significantly reduced the width of potential hairs.^16^ Conversely, inhibiting DKK1 appears to stimulate hair growth, as evidenced by DKK1 knockout (KO) experiments, which showed an increase in the number and thickness of hair follicles, resulting in enhanced hair growth.^17^, ^18^, ^19^ Furthermore, topical application of a DKK1 inhibitor has demonstrated the ability to promote hair growth in mice.^19^ Further research is necessary to gain a comprehensive understanding of the role of DKK1 in AA and to ascertain its potential as a therapeutic target.

In a previous study, DKK1 expression was examined in patients with AA and was found to be significantly higher compared with individuals without AA.^20^, ^21^ These findings indicate the potential involvement of DKK1 in the development of AA and suggest that it could be a promising therapeutic target for hair diseases. Based on this observation, we formulated the hypothesis that DKK1 KO in ASCs could serve as an effective cell‐based therapy for AA. To test this hypothesis, we generated DKK1‐KO‐ASCs and conducted a comprehensive analysis of their cellular characteristics. Our results revealed that DKK1‐KO‐ASCs exhibited a reduced inflammatory response and proved to be more effective in treating AA compared with their non‐knockout counterparts.

## 2 MATERIALS AND METHODS

### 2.1 Cell culture

Human ASCs were isolated from subcutaneous fat obtained via liposuction, following the previously described methods.^22^ Informed consent was obtained from donors at Yonsei University College of Medicine (4‐2018‐0141). The ASCs were cultured in a‐MEM medium (Hyclone, Logan, UT) supplemented with 10% fetal bovine serum (FBS, Gibco, CA, USA) and 1% penicillin/streptomycin (Gibco). Similarly, human outer root sheath cells (ORSs) were isolated from hair follicles of consenting donors at Yonsei University College of Medicine (4‐2018‐0141). The passage 0 ORS cells were cultured for 2–3 days in a culture dish containing DPC medium (Follicle dermal papilla cell growth medium with growth medium supplement mix, PromoCell, Heidelberg, Germany) with 1% penicillin/streptomycin (Gibco), after which proliferating cells were subcultured with ORS medium. The ORS cells were cultured in EpiLife medium (Gibco) supplemented with EpiLife™ Defined Growth Supplement (Gibco) and 1% penicillin/streptomycin (Gibco). For all experiments, ASCs and ORSs were maintained at 37°C in a humidified 5% CO_2_ incubator.

### 2.2 Plasmid construction

To design guide sequences, the RGEN and DeepSpCas9 websites were utilised, and gRNA sequences were selected for DKK1‐KO‐ASC. For the selected sequences, ACC was added to the 5′ end of the forward primer, followed by G, regardless of the sequence. For the reverse primer, AAC was added to the 5′ end, and C was added to the 3′ end. The guide RNA vector, px552, was cut using SapI (BioLabs, MA, USA), and a single band was confirmed after gel extraction. Primers were prepared by annealing with PNK (BioLabs). The prepared vector and primers were mixed and ligated using T4 ligase before being transformed into E. coli cells. Polymerase chain reaction (PCR) was performed, and the correct size band was confirmed. The final sequence was confirmed through sequencing. The confirmed DNA was then inoculated into LB agar and cultured before being extracted using DNA maxi prep (Qiagen, Hilden, Germany). The Crisper‐Cas9 vector and px552 vector were provided by Dr. HB Kim's laboratory at Yonsei University.

### 2.3 Electroporation

To prepare the necessary vectors in a gRNA to Cas9 ratio of 1:2, 3 μg of gRNA and 6 μg of Cas9 plasmids were added to each tube containing 5 × 10^5^ ASCs. The Neon transfection system (Invitrogen, MA, USA) was set up by retrieving the E2 buffer, R buffer, cuvette and pipette/tip set (specific to Neon), and turning on the device. The E2 and R buffers were kept on ice, and 3 mL of E2 buffer was added to the cuvette. The device was set up with the following parameters (1150 v, 30 ms, 2 pulse). After transfection, the cells were on the plate and placed in a CO_2_ incubator. The efficiency of transfection is observed by monitoring the red fluorescence signal at 24 and 48 h. The medium is changed every other day starting from 2 days post‐transfection. After 6–7 days, the cells are treated with puromycin (4 μg/mL) for 22 h and then the medium is replaced with fresh medium. The DKK1‐KO‐ASCs are then cultured for an additional 8–10 days.

### 2.4 T7E1 assay

Genomic DNA was extracted from ASCs and DKK1‐KO‐ASCs using a standard method. The target region of the DKK1 gene was amplified by PCR using primers specific to the target sequence. The purified PCR product was denatured by heating it to 95°C for 5 min and then slowly cooled to room temperature over 30 min. This step is important for generating single‐stranded DNA suitable for T7 endonuclease I (T7E1, BioLabs) digestion. A reaction mixture was prepared containing 200 ng of denatured PCR product, 1 μL of 10 × NEB buffer 2 and 0.45 μL of T7E1 enzyme. The mixture was then incubated at 37°C for 30 min. The resulting product was analysed by running the digested PCR products on an agarose gel. The presence of cleavage bands on the gel indicates the presence of mutations in the PCR product.

### 2.5 Cell growth assay

For a cell growth assay, either control ASCs or DKK1‐KO‐ASCs were seeded in 12‐well plates at a density of 5 × 10^3^ cells per well and incubated for 4 days. The cells were then trypsinized, stained with trypan blue (Sigma‐Aldrich, MO, USA) and counted daily using a haemocytometer. For a cell growth assay, human ORS cells were seeded in collagen‐coated 12‐well plates at a density of 2 × 10^4^ cells per well and treated with rhDKK1 the following day. Cell counting was performed four days after treatment. The cells were then trypsinized, stained with trypan blue (Sigma‐Aldrich, MO, USA) and counted daily using a haemocytometer.

### 2.6 Transwell migration assay

The control ASCs or DKK1‐KO‐ASCs were seeded into 60 mm plates at a density of 1.5 × 10^4^ cells/well and starved for 1 day. The cells were then suspended in a serum‐free medium and seeded on the upper side of a transwell membrane insert (BD Falcon, CA, USA), which had been pre‐coated with a 1/60 dilution of Matrigel matrix (BD Falcon). Normal serum with FBS was added to the lower plate as a chemoattractant. The cultures were incubated for 1 day to allow for transwell migration. The inserts were then removed, and the upper surface was cleaned using cotton swabs and washed with PBS to remove non‐migrating cells. The inserts were stained with a solution of 0.1% formalin and 10% crystal violet (Sigma‐Aldrich) for 30 min, and the number of cells was analysed under a Nikon ECLIPSE Ts2 fluorescence microscope. Multiple images were acquired per insert, and the average cell count was calculated.

### 2.7 RNA extraction, quantitative RT‐PCR and QPCR array

Total RNA was extracted from primary ASCs, DPCs and whole back skin tissue using Trizol reagent (Invitrogen), and cDNA synthesis was performed using oligodT and the HelixCriptTM Thermo Reverse Transcription System (Nanohelix, WI, USA) according to the manufacturer's instructions. SYBR (Takara, Kusatsu, Japan) was used for quantitative PCR reactions. For the QPCR array, total RNA was extracted from control ASCs or DKK1‐KO‐ASCs and subjected to cDNA synthesis using the same method as mentioned above. QPCR reactions for growth factors and WNT pathway‐related genes were conducted using the RT^2^ First Strand cDNA Synthesis Kit (Qiagen). The primers used are described in Table S1.

### 2.8 X‐gal staining for cellular senescence

To assess cellular senescence, a β‐galactosidase staining kit (Cell Signaling, MA, USA) was utilised following the manufacturer's instructions. Briefly, the cells were fixed with a fixation buffer composed of 20% formaldehyde and 2% glutaraldehyde in PBS for 10 min, then washed with PBS. The fixed cells were incubated at 37°C overnight in freshly prepared cell staining working solution, which contained potassium ferricyanide and X‐gal. The following day, the cell‐staining solution was discarded and the cells were washed twice with PBS. Blue‐coloured cells were considered senescent based on observation under a light microscope (Nikon ECLIPSE Ts2, Tokyo, Japan).

### 2.9 Purification of conditioned medium

To purify the conditioned medium of control ASCs or DKK1‐KO‐ASCs, the cells were cultured at a density of over 90% and incubated with serum‐free medium for 1–2 days. The medium was then centrifuged at 4°C using a VIVASPIN 20 filter (Sartorius, Goettingen, Germany) at 3000 rpm for 1 h.

### 2.10 In vitro mimic AA

ORS cells were seeded at a density of 0.5 × 10^4^ cells/well in 6‐well collagen‐coated plates and incubated for 2–3 days. After treatment with interferon‐γ (INFγ, 100 ng/mL, PeproTech, NJ, USA) and poly(I:C) (10 μg/mL, Invivogen, CA, USA) for 24 h, the cells were treated with a conditioned medium containing 20% ASCs or 20% DKK1‐KO‐ASCs and cultured for an additional 3–4 days. To assess cell growth, the cells were trypsinized, stained with trypan blue (Sigma‐Aldrich) and counted each day using a haemocytometer.

### 2.11 Ex vivo mimic AA

The isolated vibrissae hair follicles of mice and human hair follicles were treated with a specific medium containing INFγ (100 ng/mL) and poly(I:C) (10 μg/mL) for 18 h. After that, the conditioned medium of 20% control ASCs or 20% DKK1‐KO‐ASCs was added and the cultures were maintained for 1.5 days, and the length growth was measured.

### 2.12 Haematoxylin/eosin staining and immunofluorescence staining

For Haematoxylin and Eosin (H&E) staining, paraffin sections were dewaxed using xylene for 30 min, hydrated in 100%, 90%, 80% and 70% ethanol and then dipped into Mayer's Haematoxylin (Sigma‐Aldrich) for 8 min. The sections were rinsed in water for 1 min, then dipped into Eosin Y (Sigma‐Aldrich) for 30 s. Afterward, the sections were dehydrated with 70%, 80%, 90% and 100% ethanol, washed with fresh xylene for 30 min, dried and mounted with a mounting medium.

Immunofluorescence staining of paraffin‐embed sections was performed using standard protocols. Briefly, paraffin sections were dewaxed using xylene for 30 min and hydrated in 100%, 90%, 80% and 70% ethanol. Antigen retrieval was performed by boiling the sections in antigen retrieval buffer (Dako, CA, USA) using a microwave for 2 min and 20 s. The sections were then treated with primary antibodies, including mouse DKK1 antibody (1:300) (Santa Cruz, TX, USA), rabbit PDGFRA antibody (1:300) (BIOSS, MA, USA), rabbit CD34 antibody (1:300) (Abcam, IA, USA), rabbit phospho‐STAT3 antibody (1:300) (Cell Signaling Technology, MA, USA), rabbit CD4 antibody (1:300) (BIOSS, MA, USA), mouse CD8 antibody (1:300) (Santa Cruz), overnight at 4°C. The sections were then incubated with secondary antibodies, Alexa Fluor 488 goat anti‐mouse IgG or Alexa Fluor 594 goat anti‐rabbit IgG (Invitrogen), for 1 h at room temperature. Finally, the slides were mounted with VECTASHIELD (Vector Laboratories, CA, USA) with mounting medium containing DAPI. Immunofluorescence staining was imaged using a Nikon ECLIPSE Ts2 fluorescence microscope.

### 2.13 Inhibitor treatment

For STAT3 inhibition, ASC cells were seeded at a density of 5 × 10^4^ in 6‐well plates, and 10 μM Stattic (a STAT3 inhibitor, Sigma‐Aldrich) was applied for 14 h. INFγ (1 μg/mL) was administered for 20 min, and the cells were then immunostained using a phospho‐STAT3 antibody.

For NF‐kB inhibition (Figure S5), ASC cells were seeded at a density of 5 × 10^4^ in 6 well plates and 5 μM dexamethasone (Sigma‐Aldrich) was treated for 16 h. rhDKK1 (5 ng/mL) was treated for 30 min and immunostained by phospho‐NF‐kB antibody. For NF‐kB inhibition (Figure S6), ASCs or DKK1‐KO‐ASCs were seeded at a density of 5 × 10^4^ in 6 well plates and rhDKK1 (5 ng/mL) was treated for 30 min and immunostained by phospho‐NF‐kB antibody.

### 2.14 DKK1 in situ hybridization

DKK1 in situ hybridization was performed following the protocol provided by Advanced Cell Diagnostics (RNAscope® Intro Pack 2.5 HD reagent kit‐RED, cat #: 322373: RNAscope® probe‐Mm‐DKK1, cat #: 402521).

### 2.15 TUNEL assay

The TUNEL assay was performed following the protocol provided by Abcam (cat #: ab66110). ORS cells were seeded in a 6‐well plate and treated with INFγ (100 ng/mL) and poly(I:C) (10 μg/mL) to induce an AA‐like condition. After 18 h, the cells were treated with conditioned media (CM) of control ASCs or DKK1‐KO‐ASC, and 72 h later, the TUNEL assay was conducted.

### 2.16 Animal experiment in the AA model

The mice were maintained and anaesthetised according to a protocol approved by the US Pharmacopoeia and the Institutional Animal Care and Use Committee of Yonsei University (IACUC‐202302‐1636‐01). The animal model system for AA was established by administering lymph node cells from the AA model.^23^, ^24^ Four months later, 4 × 10^5^ control ASCs or DKK1‐KO‐ASC were intravenously administered into the tail vein of the AA mice. The mice were observed for a period of 3 weeks following the administration. At the end of the 3 weeks, the mice were sacrificed, and samples were collected for H&E staining and immunofluorescence staining. DKK1 neutralising antibody was subcutaneously administered once at a dose of 100 μg per mouse. The mice were observed for a period of 4 weeks following the administration. At the end of the 4 weeks, the mice were sacrificed, and samples were collected for H&E staining and immunofluorescence staining.

### 2.17 ELISA analysis

To analyse hDKK1 protein levels, an ELISA kit (R&D Systems, MN, USA) was utilised. ASC cells were seeded at a density of 3 × 104 in 12 well plates and treated with INFγ (0.5 μg/mL and 1 μg/mL) on the following day. After a 3‐day incubation period, the culture supernatant was harvested and diluted 10‐fold. The diluted samples were subsequently subjected to DKK1 level analysis using the ELISA kit. Similarly, ASC cells and DKK1‐KO‐ASC cells were seeded at a density of 3 × 104 in 12 well plates. Following a 3‐day culture, the culture supernatant was collected and diluted 10‐fold. The diluted samples were then analysed for DKK1 levels utilising the ELISA kit.

### 2.18 Next generation sequencing (NGS) analysis

Total RNA of the control ASCs or DKK1‐KO‐ASCs was extracted with Trizol Agent (Invitrogen). Each group of total RNA was prepared with three independent experiments. Extracted RNA samples were sent to Macrogen (Seoul, Korea) for NGS analysis. Preparation and construction of these samples were performed using TruSeq Stranded mRNA Library Prep Kit (Illumina, CA, USA). Paired‐ends transcriptome analysis was performed and trimmed reads were mapped using hierarchical indexing for spliced alignment of transcripts2, (HISAT2, MA, USA). After that, transcript assembly was done using StringTie (V2.2.0, MA, USA) and expression profile was obtained. Fragments Per Kilobase of transcript per Million (FPKM) were calculated and used for differentially expressed genes (DEGs) analysis with DESeq package (DESeq2). Fold change ≥ |1.5|, *p* value <0.05 were set up for DEGs. To confirm the correlation of gene expression from each sample, heatmap of two‐way hierarchical clustering was performed using RStudio. To visualise the distribution of gene expression and DEGs, volcano plot was plotted using the DESeq package.

### 2.19 Gene set enrichment analysis (GSEA)

Pre‐ranked GSEA analysis was performed with NGS data from DKK1‐KO‐ASC and control ASC. Whole gene expression data was ranked by its fold change and converted to RNK file format. To elucidate well‐defined biological properties, GMT files were used from the GSEA molecular signatures database (GSEA/msigdb, Broad Institute, UC San Diego, USA). HP:0001596 was used to investigate the involvement of alopecia with DKK1‐KO‐ASC for GSEA analysis in GMT format. Gene sets of Gene Ontology Biological Process (GOBP) were also used for GMT, specifically related to inflammation and immune response.

### 2.20 Statistical analysis

All experiments were performed at least three times using independent cultures. Data are presented as mean ± standard error. Means were compared using the student's *t*‐test. A confidence level of less than 0.05 was considered statistically significant for all tests.

## 3 RESULTS

### 3.1 DKK protein is highly expressed in ASCs of AA

**FIGURE 1:**
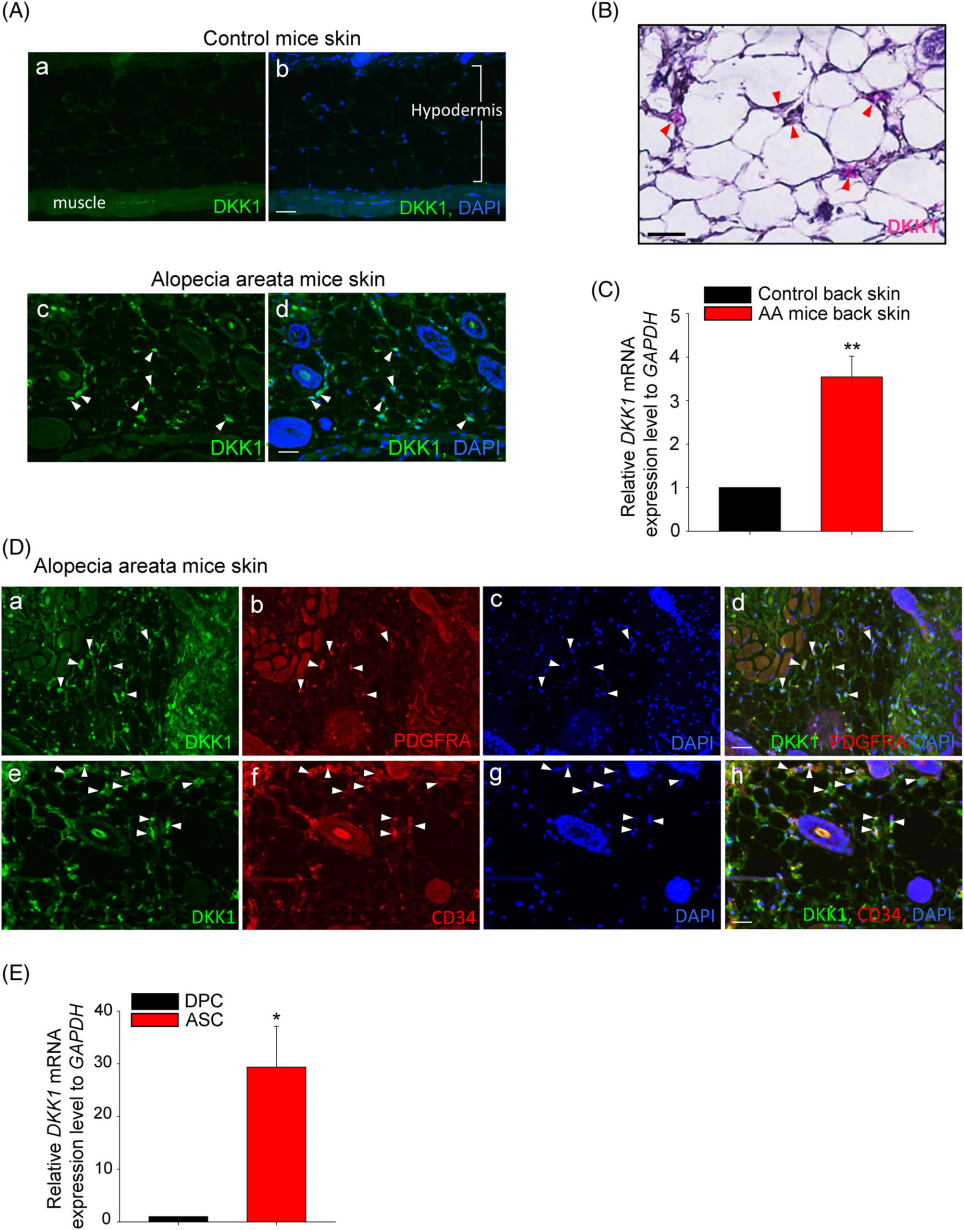
DKK expression in mouse alopecia areata (AA). (A) The expression of DKK1 was significantly elevated in the skin tissue of AA mice (arrowheads, indicated by green). (A and B) Control mice skin tissue and (C and D) AA mice skin tissue. Scale bars indicate 10 μm. (B) The mRNA expression of DKK1 was assessed through in situ hybridization, and it appeared as pink (DKK1 mRNA, arrowheads) staining between adipocytes in the skin of AA mice. Haematoxylin (purple) was counterstained. (C) The mRNA expression level of DKK1 is high in AA mice back skin tissue. (D) DKK1 protein is specifically expressed in ASCs, which are also stained positive for the ASC marker PDGFRA (A–D) and CD34 (E–H) in AA mice skin tissue. Scale bars indicate 10 μm. (E) The mRNA expression level of DKK1 was evaluated in human DPCs and ASCs and significantly higher in ASCs. *p < 0.05, **p < 0.01. Three independent experiments were conducted for all data points. Error bars indicate the S.E.M.

To investigate the potential correlation between high expression of DKK1 and hair loss, the expression of DKK1 was analysed in the scalp tissue of human patients with AA (Figure S1). DKK1 protein was found to be highly expressed in the perifollicular region of the scalp tissue in AA patients. In contrast, DKK1 expression was significantly lower in normal human scalp tissue (Figure S1A). Immunostaining using a PDGFRA antibody, which is a marker for ASCs,^25^ was performed to identify the cells expressing DKK1 in the skin tissue. DKK1 was found to co‐localise and co‐express with PDGFRA in the skin tissue (Figure S1B). The protein expression of DKK1 was also higher in the skin tissues of an AA animal compared with those in the control group (Figure 1A). As DKK1 is a secreted protein, we investigated the mRNA expression of DKK1 in ASCs using in situ hybridization. DKK1 mRNA was observed in pink staining between adipocytes in AA mouse skin (Figure 1B). In addition, the relative mRNA level of DKK1 is high in AA mice skin (Figure 1C). Further analysis of DKK1 protein expression revealed its high expression in ASCs of adipose tissue, as marked by PDGFRA and CD34 (Figure 1D). As DKK1 is reportedly expressed in DPCs,^18^ we compared the mRNA expression of DKK1 between ASCs and DPCs (Figure 1E). Expression of DKK1 was significantly higher in human ASCs compared with human DPCs (Figure 1E). These findings suggest that expression of DKK1 in ASCs is associated with hair loss in AA.

### 3.2 DKK1 expression is secreted from ASCs via STAT3 pathway

**FIGURE 2:**
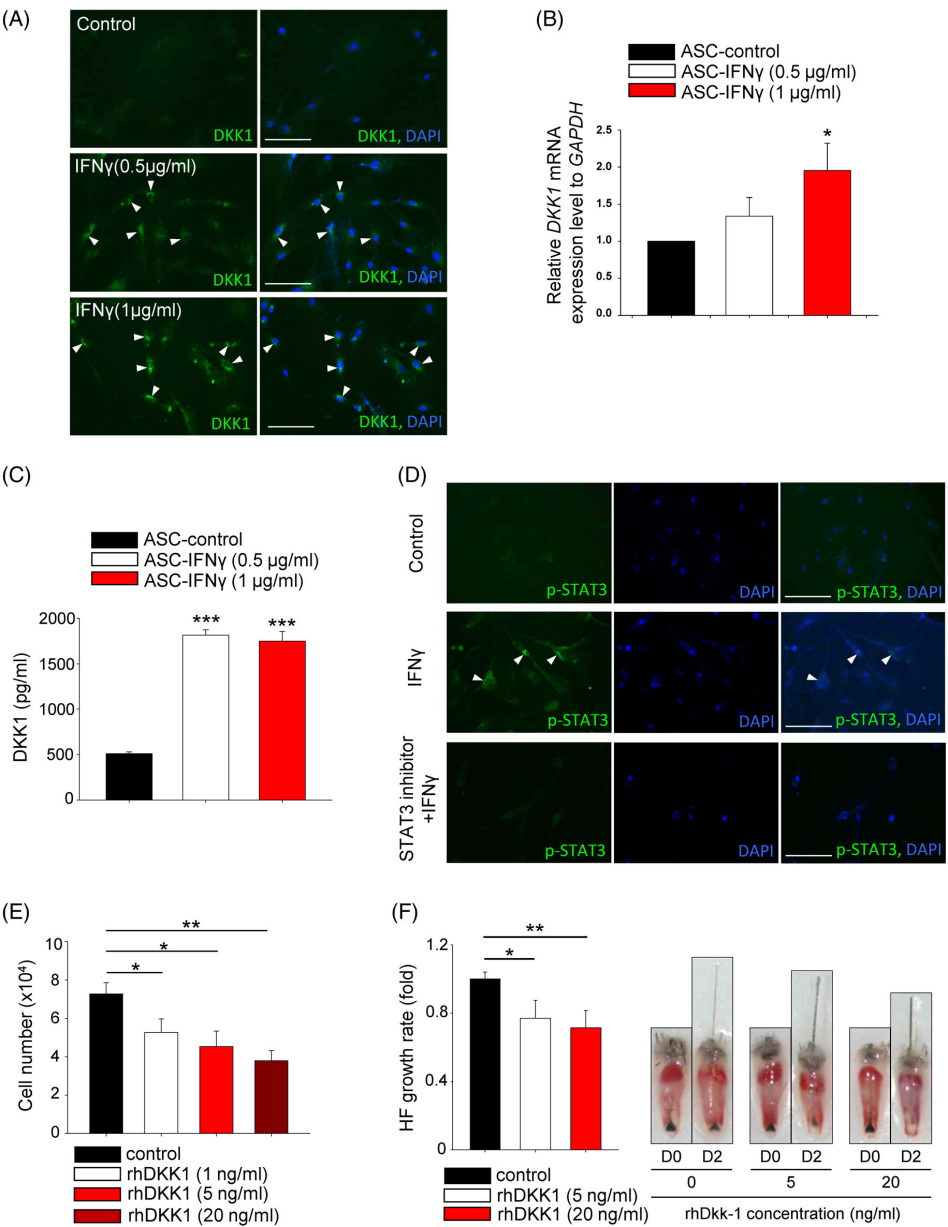
DKK1 expression is upregulated and secreted by ASCs in an AA‐mimic environment. (A) The protein level of DKK1 in ASCs was increased by IFNγ treatment (arrowheads). Scale bars indicate 10 μm. (B) The mRNA expression level of DKK1 was elevated after IFNγ treatment in ASCs. (C) The secreted DKK1 from ASCs was measured using the ELISA method and also increased after INFγ treatment. (D) The activation of STAT3 was evaluated using immunostaining with p‐STAT3. INFγ treatment increased the phosphorylation of STAT3 (arrowheads) and STAT3 inhibitor suppressed phosphorylation of STAT3. Scale bars indicate 10 μm. (E) The inhibitory effect of human recombinant DKK1 (rhDKK1) treatment on the proliferation of ORS cells was evaluated. (F) The inhibitory effect of rhDKK1 treatment on hair growth was also evaluated in an organ culture model using mouse vibrissa hair follicle (HF). More than 10 samples were analysed per group. *p < 0.05, **p < 0.01, ***p < 0.001. Three independent experiments were conducted for all in vitro data points. Error bars indicate the S.E.M.

To investigate whether or not ASCs secrete DKK1 following the INFγ treatment, DKK1 expression was observed in both the mRNA and protein levels (Figure 2A, B). In the ELISA assay, INFγ treatment resulted in an increased secretion of DKK1 from ASCs (Figure 2C). We further investigated the involvement of other cytokines (i.e., IL‐6, IL‐1α, TNF‐α) in DKK1 expression and found that IL‐6 increased the protein expression of DKK1 (Figure S2).

The JAK–STAT pathway is known to play a critical role in the pathogenesis of AA, and DKK1 expression is reportedly elevated through STAT3 pathway.^26^, ^27^, ^28^, ^29^ Therefore, phosphorylated STAT3 was assessed after INFγ treatment and pharmacological inhibition of STAT3 by Stattic. The results revealed the involvement of STAT3 pathway in DKK1 secretion (Figure 2D).

To investigate whether or not secreted DKK1 from ASCs could directly harm hair follicles, ORS cells (i.e., highly susceptible in AA conditions) were treated with recombinant human DKK1 (rhDKK1) protein. As a result, the growth of ORS cells was significantly decreased after 1–20 ng/mL rhDKK1 treatment (Figure 2E). Furthermore, rhDKK1 treatment also significantly inhibited hair follicle growth in the organ culture model (Figure 2F).

### 3.3 DKK1 neutralising antibody restores hair growth in the AA model

**FIGURE 3:**
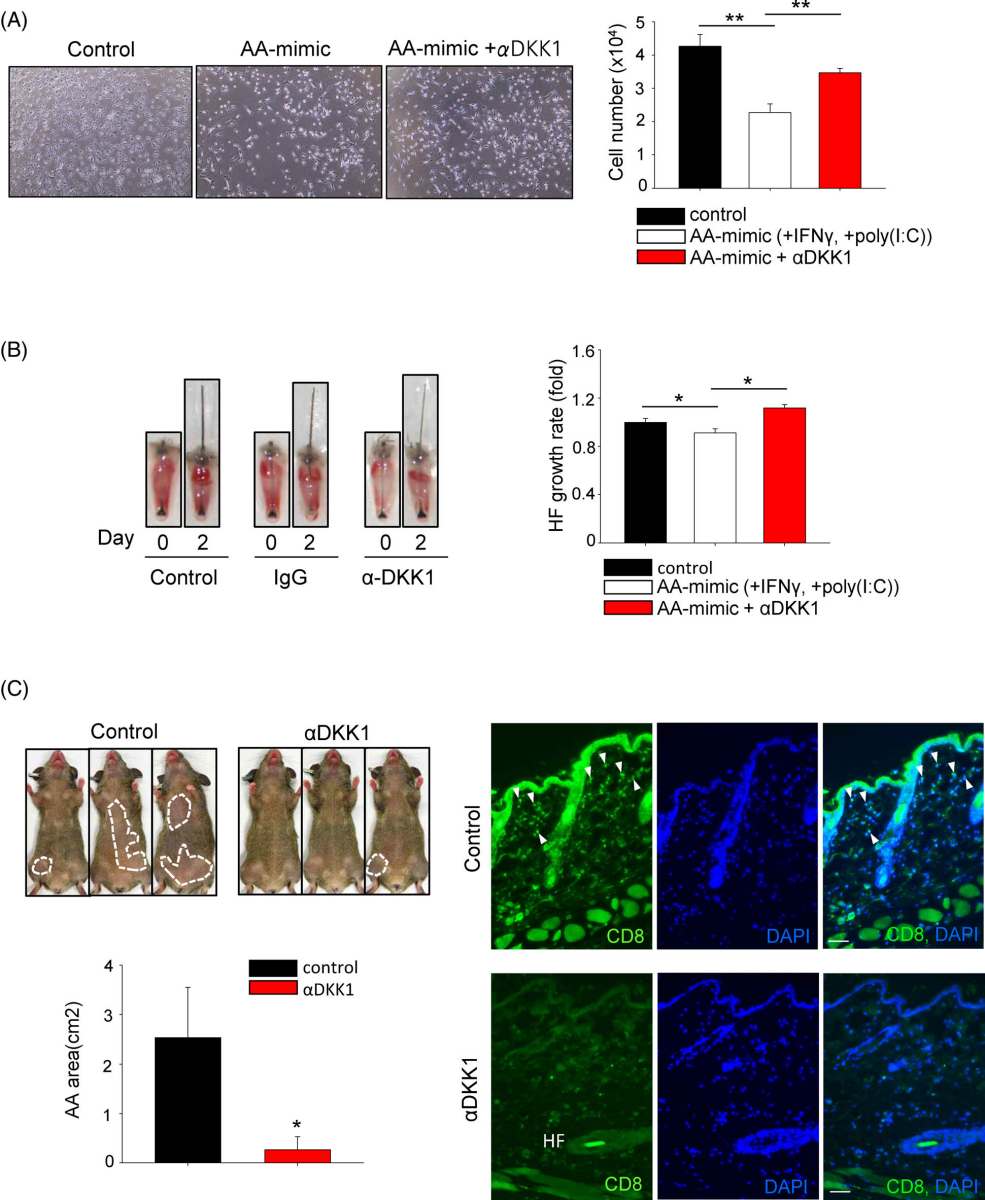
The DKK1 neutralising antibody (αDKK1) restores hair loss in the AA model. (A) Treatment with IFNγ and poly(I:C) induced cell death in ORS cells, and the impact of αDKK1 treatment on cell death recovery was assessed at 72 h. Three independent experiments were conducted for all data points. (B) The effect of αDKK1 was also evaluated in an ex vivo AA model using mouse vibrissa hair follicles (HF). αDKK1 treatment resulted in increased hair length in organ culture. More than 10 samples were analysed per group. (C) The therapeutic efficacy of αDKK1 was assessed in an AA animal model. Following subcutaneous administration of αDKK1, the area of hair loss in AA was markedly reduced for 4 weeks, and CD8^+^ cells (arrowheads) were reduced in the skin. Three mice were analysed per group. *p < 0.05, **p < 0.01. Error bars indicate the S.E.M. Scale bars indicate 10 μm.

Previous studies have created in vitro and ex vivo models of AA by treating with IFNγ and poly(I:C) to mimic the AA environment.^24^, ^30^ Therefore, we made in vitro AA‐mimic model using ORS cells, and DKK1 neutralising antibody restored the ORS cell apoptosis (Figure 3A).

Furthermore, the recovery effect of DKK1 neutralising antibody was observed in an organ culture model. Treatment with DKK1 neutralising antibody restored the hair loss induced by IFNγ and poly(I:C) (Figure 3B). The recovery effect of DKK1 neutralising antibody treatment was also observed in an animal model. Following a single subcutaneous administration of DKK1 neutralising antibody, a gradual recovery of hair loss was observed for 4 weeks (Figure 3C). In addition, CD8^+^ cells were decreased in DKK1 neutralising antibody treatment. Collectively, these results suggest that regulating the levels of DKK1 can restore AA.

### 3.4 Establishment of DKK1‐KO‐ASCs

**FIGURE 4:**
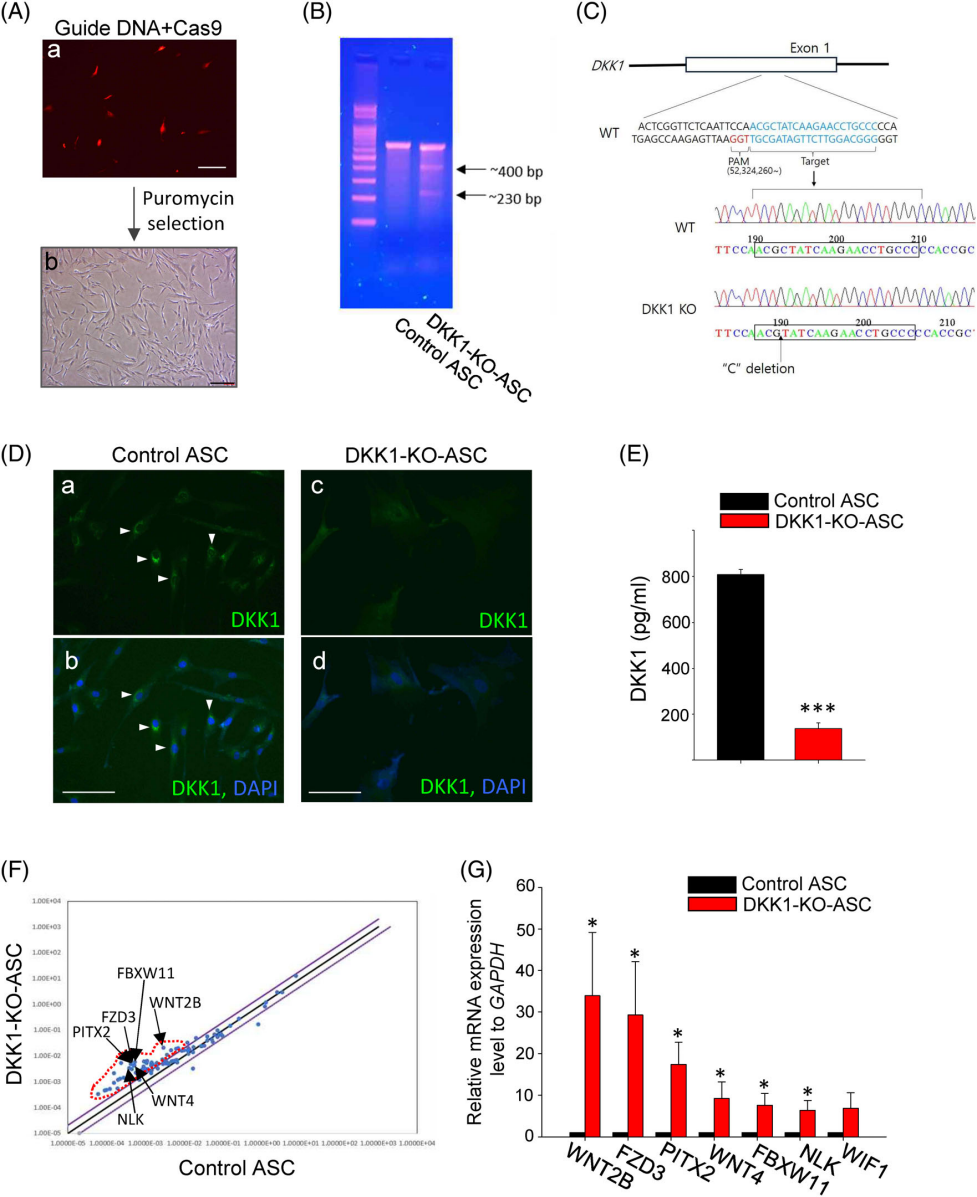
Establishment of DKK1‐KO‐ASCs. (A) Following the transfection of the Cas9 vector and guide RNA vector, puromycin treatment was administered, resulting in the successful acquisition of DKK1‐KO‐ASCs. Scale bars indicate 10 μm. (B) A T7E1 assay was conducted using genomic DNA isolated from DKK1‐KO‐ASCs. (C) Sanger sequencing was performed using genomic DNA obtained from DKK1‐KO‐ASCs, revealing mutations at the expected guide region. The results confirmed the presence of mutation in the EXON 1 region of the DKK1 gene in DKK1‐KO‐ASC cells. (D) Immunostaining analysis revealed that DKK1 protein expression was hardly detectable in DKK1‐KO‐ASCs (DKK1: arrowheads). Scale bars indicate 10 μm. (E) The secretion of DKK1 from Control‐ASC and DKK1‐KO‐ASCs was quantified using ELISA. (F) A QPCR array was conducted to analyse WNT pathway‐related genes in both control ASCs and DKK1‐KO‐ASCs, revealing that the WNT pathways are activated by DKK1‐KO. (G) The expression of top‐notch genes was confirmed through QPCR reactions. Three independent experiments were conducted for all data points. *p < 0.05, ***p < 0.001. Error bars indicate the S.E.M.

DKK1 is highly expressed in ASCs, and neutralising DKK1 has shown potential for AA treatment. Therefore, we generated DKK1‐KO‐ASCs and applied them to treat AA. A specific guide sequence targeting the EXON 1 region was selected, and cloning was performed followed by the transfection into ASCs using the electroporation method. After transfection and puromycin selection, strong expression of the red fluorescence signal in the Cas9 vector was confirmed (Figure 4A). The occurrence of mutations within the designed region in the established cell line was demonstrated using the T7E1 assay (Figure 4B). Genomic DNA sequencing of the established cell line confirmed random mutations at the targeted site (Figure 4C). The DKK1 protein level was almost undetectable, indicating a nearly 95% reduction in DKK1 expression (Figure 4D), and the secreted DKK1 level from the DKK1‐KO‐ASCs was significantly low (Figure 4E).

Since DKK1 is a negative regulator of the WNT signalling pathway, we investigated the effect of DKK1 KO on the expression of WNT‐related genes using a qPCR array. Compared with the control group, DKK1‐KO‐ASCs showed increased expression of WNT‐related genes (Figure 4F), and the differential expression of selected genes was confirmed through qPCR analysis (Figure 4G).

Compared with control, DKK1‐KO‐ASCs exhibited enhanced growth and mobility (Figure S3A,B). Furthermore, cellular senescence was significantly reduced in DKK1‐KO‐ASCs compared with ASCs (p11–p12) (Figure S3C). In addition, we investigated whether DKK1‐KO in ASCs exhibits enhanced hair growth‐promoting effects compared with naïve ASCs. We observed an accelerated transition from telogen to anagen in mice (Figure S4A), and conditioned medium obtained from DKK1‐KO‐ASCs significantly increased hair follicle growth in both mouse (Figure S4B) and human hair organ cultures (Figure S4C). These findings suggest that DKK1‐KO‐ASCs are promising for cell therapy.

### 3.5 Analysis of global gene expression

**FIGURE 5:**
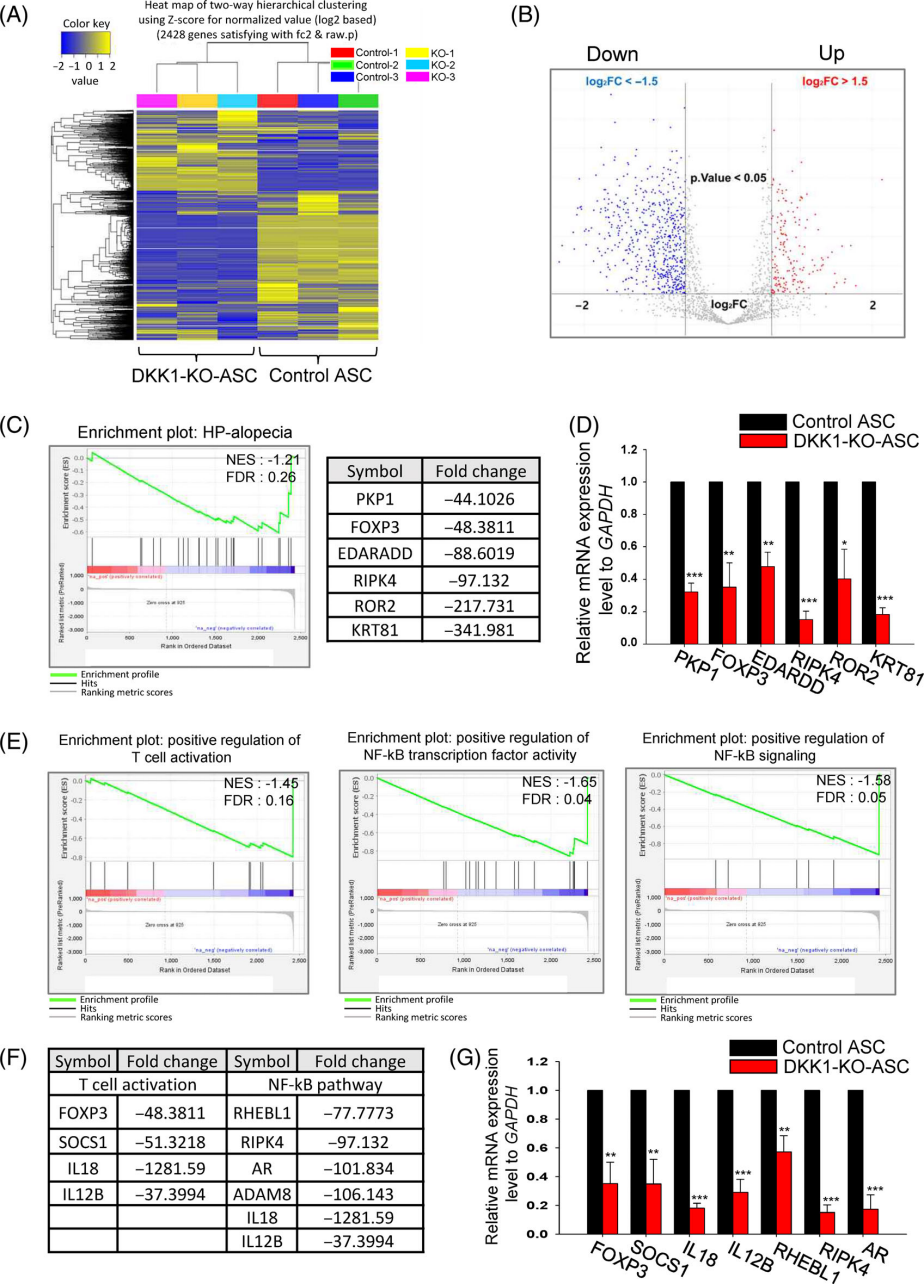
Global gene expression analysis of DKK1‐KO‐ASCs using next‐generation sequencing. (A) A heatmap was generated using two‐way hierarchical clustering with Z‐scores for normalised values. Clustering was performed for significant genes based on the similarity of expression patterns. (B) A volcano plot was created, with up‐regulated DEGs highlighted in red and down‐regulated genes in blue. Genes with a fold change >|1.5| and a *p* value <0.05 were omitted for improved visualisation. (C) Gene Set Enrichment Analysis (GSEA) of KEGG pathways revealed a significant downregulation of the alopecia‐related pathway in DKK1‐KO‐ASCs. (D) The expression levels of the top‐ranked six genes were assessed via QPCR, and they were significantly downregulated compared with control‐ASCs. (E, F) T‐cell activation and NF‐kB‐related pathway was also downregulated in DKK1‐KO‐ASCs. (G) The expression levels of the top‐ranked genes in DKK1‐KO‐ASCs were significantly decreased compared with control‐ASCs. *p < 0.05, **p < 0.01, ***p < 0.001. Three independent experiments were conducted for all QPCR data points. Error bars indicate the S.E.M.

To elucidate the altered gene expression of DKK1‐KO‐ASCs, we performed a global gene expression analysis using next‐generation sequencing. DEG analysis revealed significant changes in global gene expression between DKK1‐KO‐ASCs and control ASCs (Figure 5A,B). In DKK1‐KO‐ASCs, the expression of genes associated with hair loss was significantly decreased compared with the control group (Figure 5C). We selected the most significantly downregulated genes and confirmed their reduced expression through qPCR analysis (Figure 5D).

Moreover, genes involved in T cell activation (a key mechanism in the pathogenesis of AA) were also found to be significantly downregulated in DKK1‐KO‐ASCs (Figure 5E). The decreased expression of key genes involved in this mechanism was further confirmed by qPCR (Figure 5F,G). Also, the NF‐kB pathway is known to play a role in the pathogenesis of AA, and DKK1‐KO‐ASCs exhibited significantly lower expression of NF‐kB‐related genes compared with the control group (Figure 5E). The downregulation of key genes in the NF‐kB pathway was also confirmed by qPCR (Figure 5F,G).

We further investigated whether NF‐kB pathway is involved in DKK1‐mediated inflammation and hair loss. We assessed NF‐kB phosphorylation in naive ASCs after rhDKK1 treatment and found that DKK1 increased NF‐kB phosphorylation (Figure S5A). However, when rhDKK1 was treated with dexamethasone, a known NF‐kB inhibitor, NF‐kB phosphorylation was reduced (Figure S5A). Furthermore, we found a significant increase in the expression of NF‐kB‐related genes after rhDKK1 treatment (Figure S5B). These findings corresponded to the decreased expression of NF‐kB‐related genes observed in DKK1‐KO‐ASCs (Figure 5G). Similarly, rhDKK1 treatment did not increase NF‐kB phosphorylation in DKK1‐KO‐ASCs (Figure S6).

### 3.6 DKK1‐KO‐ASCs promote hair growth in the AA model

**FIGURE 6:**
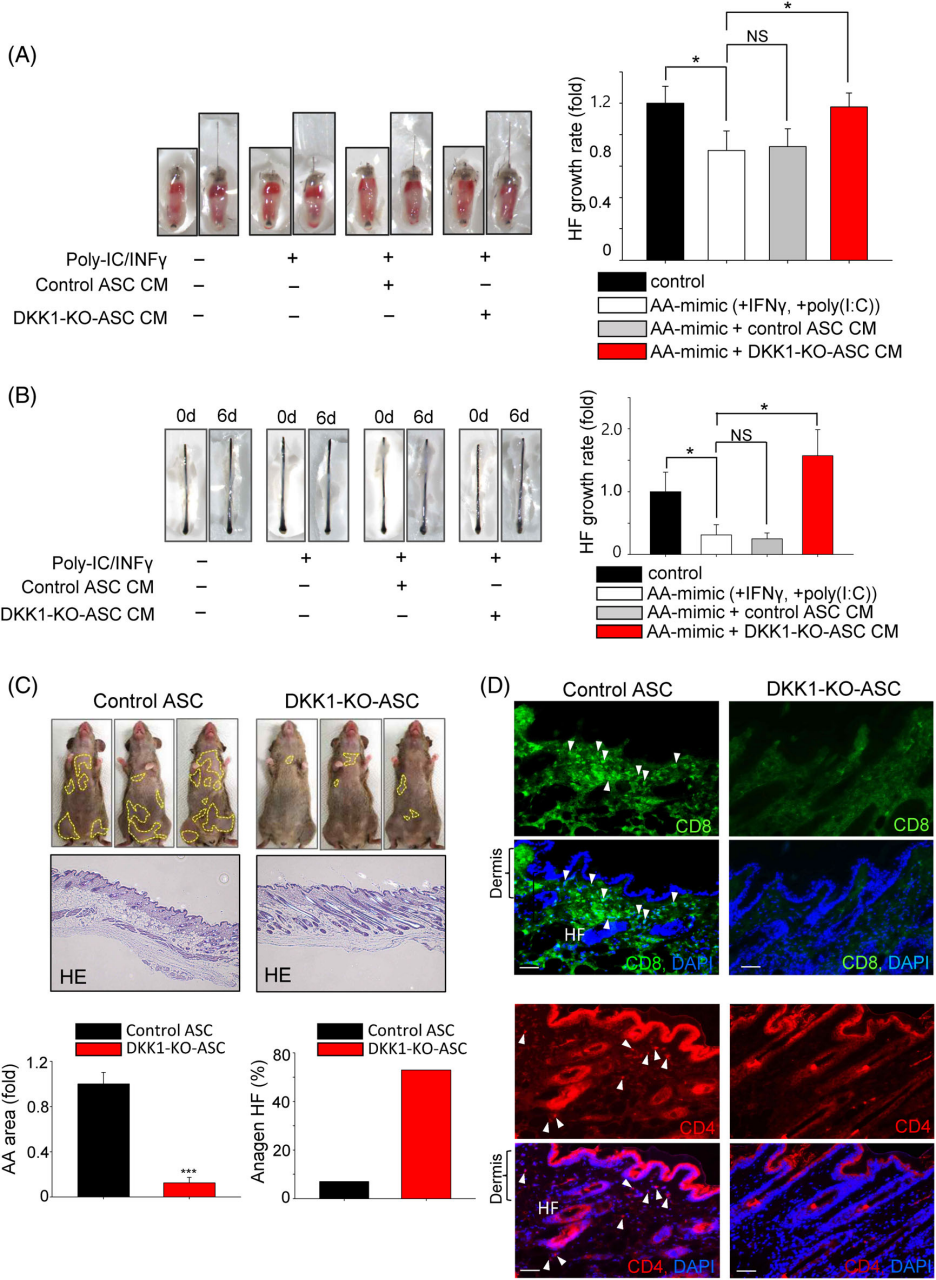
DKK1‐KO‐ASCs promote hair growth in the AA model. (A) The restorative effects of DKK1‐KO‐ASCs were assessed in an ex vivo AA model using mouse vibrissa hair follicles. Isolated mouse vibrissae hair follicles (HF) were subjected to treatment with poly(I:C) and IFNγ and then the conditioned medium (CM) from control ASCs (20%) or DKK1 KO‐ASCs (20%) was introduced. The CM treatment from DKK1‐KO‐ASCs significantly reversed the reduced hair growth induced by poly(I:C) and IFNγ. More than 10 samples were analysed per group. (B) The restorative effects of DKK1‐KO‐ASCs were also assessed in human hair follicles (HF), and the treatment with CM from DKK1‐KO‐ASCs resulted in increased hair length. More than 10 samples were analysed per group. (C) The therapeutic effectiveness of DKK1‐KO‐ASCs was assessed in the AA animal model. Following intravenous administration into the tail vein of AA mice, the area of hair loss was monitored for up to 3 weeks and the number of anagen hair follicle was evaluated. H&E staining revealed an increase in hair follicles following DKK1‐KO‐ASCs treatment. Three mice were analysed per group. (D) The expression patterns of CD4^+^ and CD8^+^ cells were assessed through immunostaining and were significantly reduced in the skin of DKK1‐KO‐ASCs‐treated mice. Scale bars indicate 10 μm. *p < 0.05. ***p < 0.001. Error bars indicate the S.E.M.

To evaluate whether DKK1‐KO‐ASCs are effective in AA treatment, we first used organ culture models using mouse vibrissa hair follicles and human hair follicles. We assessed the hair growth effect of conditioned media (CM) from DKK1‐KO‐ASCs and control ASCs. In both model systems, hair follicle growth was inhibited after the treatment with IFNγ and poly(I:C) and CM from DKK1‐KO‐ASC restored the inhibition of hair follicle growth (Figure 6A,B). Then, we created a mouse model of AA and injected DKK1‐KO‐ASC intravenously into the tail vein, observing the mice for 3 weeks. The group injected with DKK1‐KO‐ASCs showed a reduction in the area of the AA and restoration of the AA phenotype (Figure 6C). However, the control group did not show recovery of the AA phenotype (Figure 6C). Examination of skin tissues from the mice with restored hair growth revealed an increase in anagen‐phase hair follicles (Figure 6C). Furthermore, we observed a decrease in T cells, such as CD4^+^ and CD8^+^, which are known to be elevated in the vicinity of hair follicles during AA^31^ (Figure 6D).

We also investigated the restoring effects of DKK1‐KO‐ASCs in vitro using human ORS cells. Co‐treatment of IFNγ and poly(I:C) resulted in rapid cell death, but the treatment with CM of DKK1‐KO‐ASCs recovered the cell damage (Figure S7). Collectively, these results suggest that DKK1‐KO‐ASCs promote hair growth in the AA model.

## 4 DISCUSSION

DKK1 is widely recognised as a key pathogenic mediator involved in male pattern baldness. In this study, we initially examined the expression of DKK1 in the skin of mouse and human with AA and discovered a significant increase in its expression within ASCs. Subsequent treatment with recombinant DKK1 in hair follicles resulted in the induction of an inflammatory response. Conversely, the administration of a DKK1 neutralising antibody showed improvement in AA in both mice and hair organ cultures. Given the clinical evaluation of ASCs for AA treatment, we generated DKK1‐KO‐ASCs and investigated their potential enhanced effectiveness in treating AA. We further identified that DKK1‐KO in ASCs stimulated the WNT signalling pathway, promoting proliferation, while concurrently inhibiting the NF‐kB pathway to reduce inflammatory responses. To assess the comparative efficacy, we employed various AA models and observed that DKK1‐KO‐ASCs, along with their conditioned medium, showed greater effectiveness in AA treatment compared with ASCs alone.

DKK1 is a multifunctional protein expressed in various tissues, including the skin, where its expression is dynamically regulated across different compartments. It plays a crucial role in the development, cycling and regeneration of hair follicles. Specifically, DKK1 is highly expressed in the DP and contributes to maintaining the balance between the anagen and telogen of the hair cycle. During the telogen phase, DKK1 expression in the DP is upregulated, promoting follicle regression and preserving follicle quiescence.^17^, ^18^ Furthermore, DKK1 is also present in the ORS of the hair follicle, particularly in the bulge region, which harbours hair follicle stem cells. In this location, DKK1 is involved in regulating the behaviour and fate of the hair follicle stem cells, influencing their activation, proliferation and differentiation during the hair growth cycle.^16^, ^17^, ^18^, ^19^ Recent studies have examined the expression of DKK1 in patients with androgenetic alopecia and AA and have shown significantly higher levels of DKK1 compared with control subjects.^20^, ^21^ However, the spatial localization of DKK1 in these conditions has not been reported in previous studies. In our study, for the first time, we demonstrate that DKK1 is highly expressed in ASCs, where it exerts an inhibitory effect on hair growth in AA.

Under normal physiological conditions, ASCs have been found to express DKK1. However, the expression of DKK1 in ASCs can vary depending on factors, such as donor variability, age and the anatomical location of the adipose tissue source. During adipogenic differentiation of ASCs, DKK1 expression tends to decrease.^32^, ^33^ Additionally, insulin and glucocorticoids can regulate DKK1 expression to modulate the process of adipogenesis.^34^, ^35^ Interestingly, in our research, we discovered high expression of DKK1 in ASCs obtained from both patients and mice with AA. While DKK1 is primarily recognised for its role in regulating the WNT signalling pathway, emerging evidence suggests its involvement in modulating inflammatory processes.^36^ For instance, DKK1 can influence the activation and function of immune cells, including dendritic cells and T cells, which are pivotal in initiating and perpetuating inflammatory responses.^36^ Moreover, DKK1 can impact the production and release of various cytokines, such as TNFα and IL‐6, which are implicated in skin inflammation.^37^ Considering these findings, it is plausible to hypothesise that the increased secretion of DKK1 from ASCs plays a significant role in the development of AA through activating inflammatory responses.

Although our study primarily focused on the effect of DKK1 secreted from ASCs, it is worth noting that DKK1 derived from dermal fibroblasts (DFs) could also have an impact on hair follicles in the case of AA. Previous research has reported that DKK1 from DFs can influence skin colour and thickness. For example, increased expression of DKK1 by palmoplantar fibroblasts inhibits melanocyte growth and differentiation, leading to hypopigmentation in the skin on the palms and soles compared with other body areas.^38^ Additionally, DKK1 expression is significantly lower in pachydermoperiostosis fibroblasts compared with normal fibroblasts and enhanced Wnt signalling contributes to the development of pachydermia by promoting DF functions.^39^ Although we did not investigate the function of DKK1 in DFs, its expression is also increased in AA (data not shown). In addition to inhibiting melanin synthesis, DKK1 from DFs may also play an inhibitory role in hair growth in AA.

ASCs have been extensively studied for their potential to promote hair growth due to their ability to secrete growth factors and cytokines. However, their effectiveness in this regard is limited, prompting researchers to explore various strategies to enhance their hair growth effects. One such approach involves priming ASCs using hypoxia or growth factors such as EREG and PDGF.^6^, ^10^ While priming methods have the advantage of simultaneously affecting multiple genes or pathways, gene knockout offers a highly specific and precise targeting of a particular gene or pathway. Gene knockout involves a permanent modification of the genome, whereas priming typically induces transient or reversible modifications of the epigenome.^40^ Consequently, gene knockout cells may exhibit a more stable and consistent phenotype over time, whereas primed cells may lose their primed state or revert back to their original state. Therefore, we prepared ASCs with a knockout of the DKK1 gene, which acts as an inhibitor of the WNT pathway and an activator of immune reactions. By eliminating the expression of DKK1, which has been implicated in the pathogenesis of AA, we aimed to enhance the therapeutic potential of ASCs in treating AA. As expected, the use of DKK1‐KO‐ASCs showed superior effects compared with naïve ASCs in the treatment of AA.

Knockout of DKK1 in ASCs resulted in the activation of the WNT pathway, leading to increased survival and proliferation of ASCs. This activation of the WNT pathway further stimulated the secretion of growth factors, thereby promoting hair growth. Notably, RNA sequencing analysis revealed that DKK1 knockout also led to the inactivation of the T cell regulation and NF‐kB pathway (Figure 5), resulting in reduced expression of related genes such as FOXP3, SOCS1, IL18, IL12B, RIPK4 and AR. Inactivation of the NF‐kB pathway hindered the release and subsequent translocation of NF‐kB dimers, typically comprised of p50 and p65 subunits, from the cytoplasm to the nucleus. Hence, the knockout of DKK1 effectively diminished the activation of the NF‐kB pathway, thereby enhancing the anti‐inflammatory effects for the treatment of AA. By reducing the expression of these inflammatory genes, DKK1‐KO‐ASCs exhibit a potential to mitigate immune and inflammatory responses, which are implicated in the pathogenesis of AA. This dual effect of DKK1 knockout, activating the WNT pathway and inhibiting the NF‐kB pathway, shows promise in improving the treatment of AA by promoting hair growth and suppressing inflammation simultaneously.

## AUTHOR CONTRIBUTIONS

Nahyun Choi involved in conceptualization, data curation, formal analysis, investigation, methodology, validation, visualisation and writing original draft preparation. Juyeong Hwang involved in investigation, methodology, validation, formal analysis and visualisation. Doo Yeong Kim involved in NGS analysis and writing. Jino Kim involved in writing review and editing. Seung Yong Song involved in writing review and editing. Jong‐Hyuk Sung involved in conceptualization, funding acquisition, project administration, supervision, validation and writing original draft preparation.

## FUNDING INFORMATION

This research was supported by Korean Fund for Regenerative Medicine funded by Ministry of Science and ICT, and Ministry of Health and Welfare (23C0125L1, Republic of Korea).

## CONFLICT OF INTEREST STATEMENT

The authors declare no conflicts of interest.

## Supporting information


**Figure S1.** DKK expression in human alopecia areata (AA). DKK1 was found to be highly expressed in the scalp tissue of human AA (indicated by green), and it was specifically expressed in ASCs, which co‐stained with PDGFRA (an ASC marker, indicated by arrowheads). HF: hair follicle. Scale bars indicate 10 μm.

**Figure S2.** The role of inflammatory cytokines in DKK1 expression in ASCs was investigated. IL‐6 treatment resulted in an increase in DKK1 protein (green, arrowheads), whereas IL‐1α and TNF‐α did not have the same effect. Scale bars indicate 10 μm.

**Figure S3.** The effect of DKK1‐KO on cell motility and senescence of ASCs. Following the establishment of DKK1‐KO‐ASC cell lines, various aspects were assessed including proliferation (A), migration (B) and senescence (C). DKK1‐KO‐ASCs demonstrated increased proliferation and migration in comparison to control ASCs, along with reduced cellular senescence. *p < 0.05, ***p < 0.001. Three independent experiments were conducted for all data points. Error bars indicate the S.E.M.

**Figure S4.** Enhanced hair growth‐promoting effects of DKK1‐KO ASCs compared with naïve ASCs. (A) Injection of DKK1‐KO‐ASCs accelerated the transition from telogen to anagen in mice. (B, C) Treatment with conditioned medium obtained from DKK1‐KO‐ASCs significantly increased hair follicle (HF) growth in mouse (B) and human organ culture (C). More than 10 samples were analysed per group. *p < 0.05, **p < 0.01.

**Figure S5.** Activation of the NF‐kB pathway by rhDKK1 treatment. (A) Naive ASCs were treated with rhDKK1 (5 ng/mL) for 30 min and were immunostained with phospho‐NF‐kB antibody. Prior to rhDKK1 treatment, the NF‐kB inhibitor (dexamethasone) was pre‐treated, and subsequent p‐NF‐kB immunostaining was performed. Control: A–C, rhDKK1 treatment; D–F, Dexamethasone and rhDKK1 treatment; G–I. Arrowheads indicate NF‐kB activation, while arrows NF‐kB inactivation. Scale bars indicate 10 μm. (B) The expression level of NF‐kB pathway‐related genes was assessed following rhDKK1 treatment in naïve ASCs. *p < 0.05, **p < 0.01. Three independent experiments were conducted for all QPCR data points. Error bars indicate the S.E.M.

**Figure S6.** Inactivation of NF‐kB in DKK1‐KO‐ASCs. Control‐ASCs and DKK1‐KO‐ASCs were subjected to treatment with rhDKK1 (5 ng/mL) for 30 min, followed by immunostaining with a phospho‐NF‐kB antibody. Arrowheads indicate NF‐kB activation, while arrows indicate NF‐kB inactivation. Scale bars indicate 10 μm.

**Figure S7.** Effect of DKK1‐KO‐ASCs on the AA‐mimicking cells. (A) Treatment with IFNγ and poly(I:C) induced cell death in ORS cells, and the impact of DKK1‐KO‐ASC treatment on cell death recovery was also examined. Scale bars indicate 10 μm. (B) ORS cell growth in response to IFNγ and poly(I:C) was assessed at 24 and 48 h. (C) ORS cell death was evaluated using apoptosis assay. **p < 0.01, ***p < 0.001. Three independent experiments were conducted for all data points. Error bars indicate the S.E.M.

**Table S1.** Primer list used for QPCR.

## Data Availability

The data that support the findings of this study are available from the corresponding author upon reasonable request.
