# Supplementary figures and images for: Involvement of DKK1 secreted from adipose‐derived stem cells in alopecia areata
Source: Cell Prolif. 2023 Nov 22;57(3):e13562. doi: 10.1111/cpr.13562 (PMC10905327; doi:10.1111/cpr.13562)

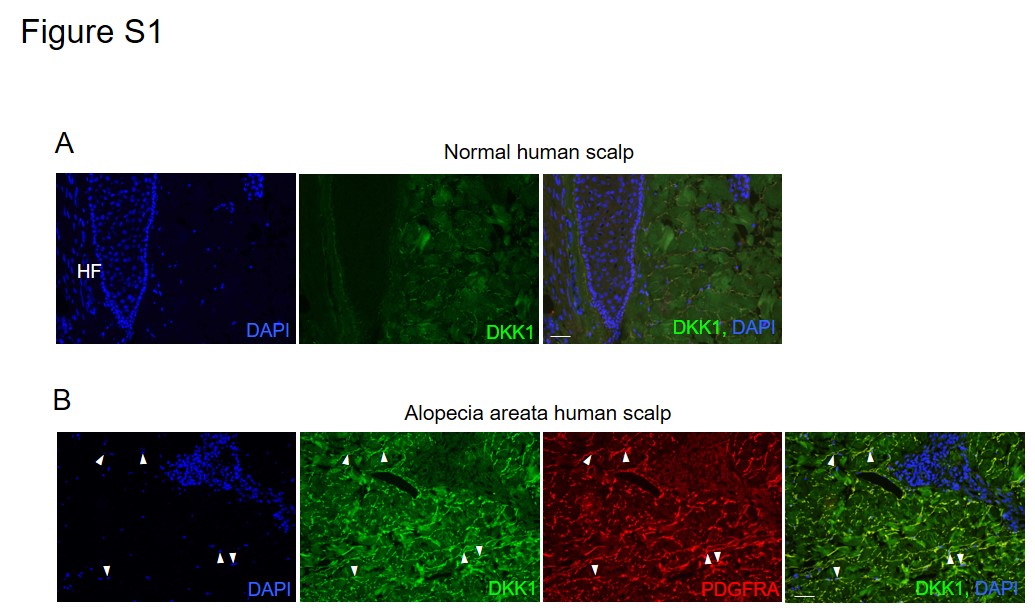

Supplement: Supplementary file 1 — Figure S1. DKK expression in human alopecia areata (AA). DKK1 was found to be highly expressed in the scalp tissue of human AA (indicated by green), and it was specifically expressed in ASCs, which co‐stained with PDGFRA (an ASC marker, indicated by arrowheads). HF: hair follicle. Scale bars indicate 10 μm. [file CPR-57-e13562-s004.jpg]

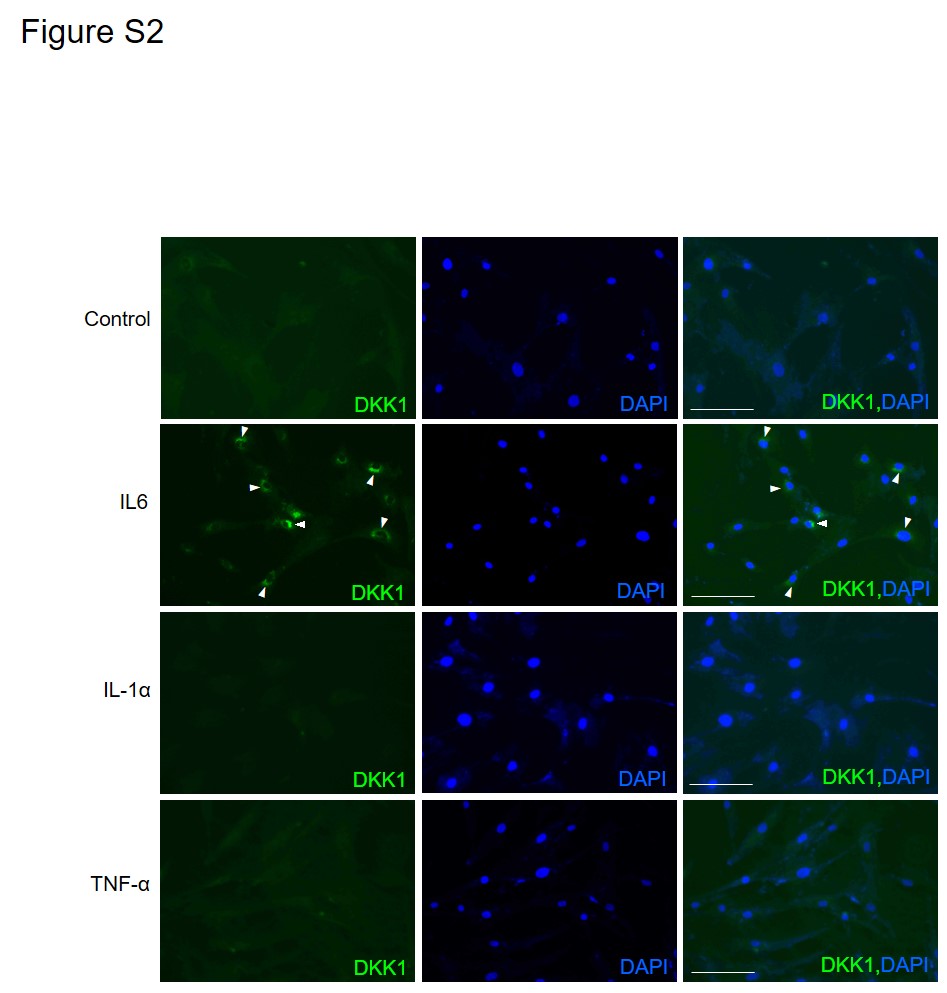

Supplement: Supplementary file 2 — Figure S2. The role of inflammatory cytokines in DKK1 expression in ASCs was investigated. IL‐6 treatment resulted in an increase in DKK1 protein (green, arrowheads), whereas IL‐1α and TNF‐α did not have the same effect. Scale bars indicate 10 μm. [file CPR-57-e13562-s007.jpg]

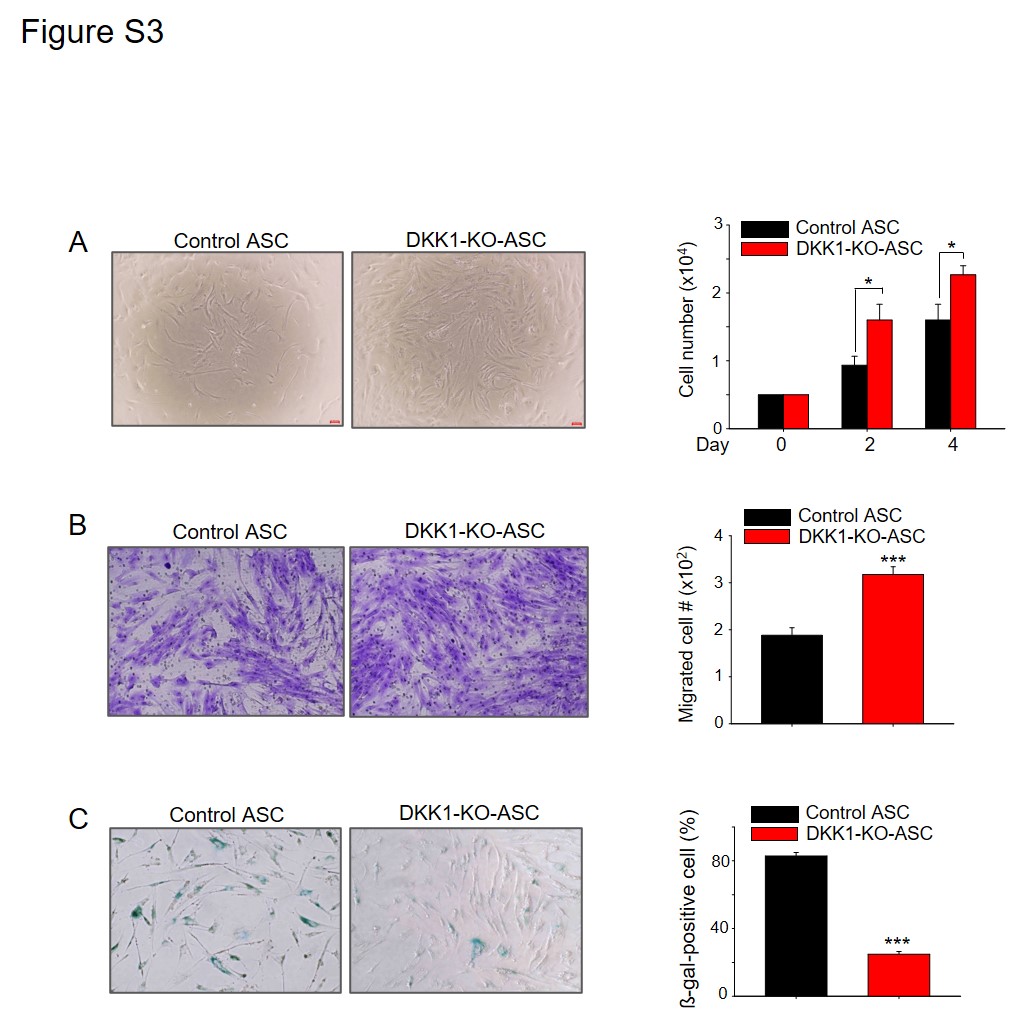

Supplement: Supplementary file 3 — Figure S3. The effect of DKK1‐KO on cell motility and senescence of ASCs. Following the establishment of DKK1‐KO‐ASC cell lines, various aspects were assessed including proliferation (A), migration (B) and senescence (C). DKK1‐KO‐ASCs demonstrated increased proliferation and migration in comparison to control ASCs, along with reduced cellular senescence. *p < 0.05, ***p < 0.001. Three independent experiments were conducted for all data points. Error bars indicate the S.E.M. [file CPR-57-e13562-s003.jpg]

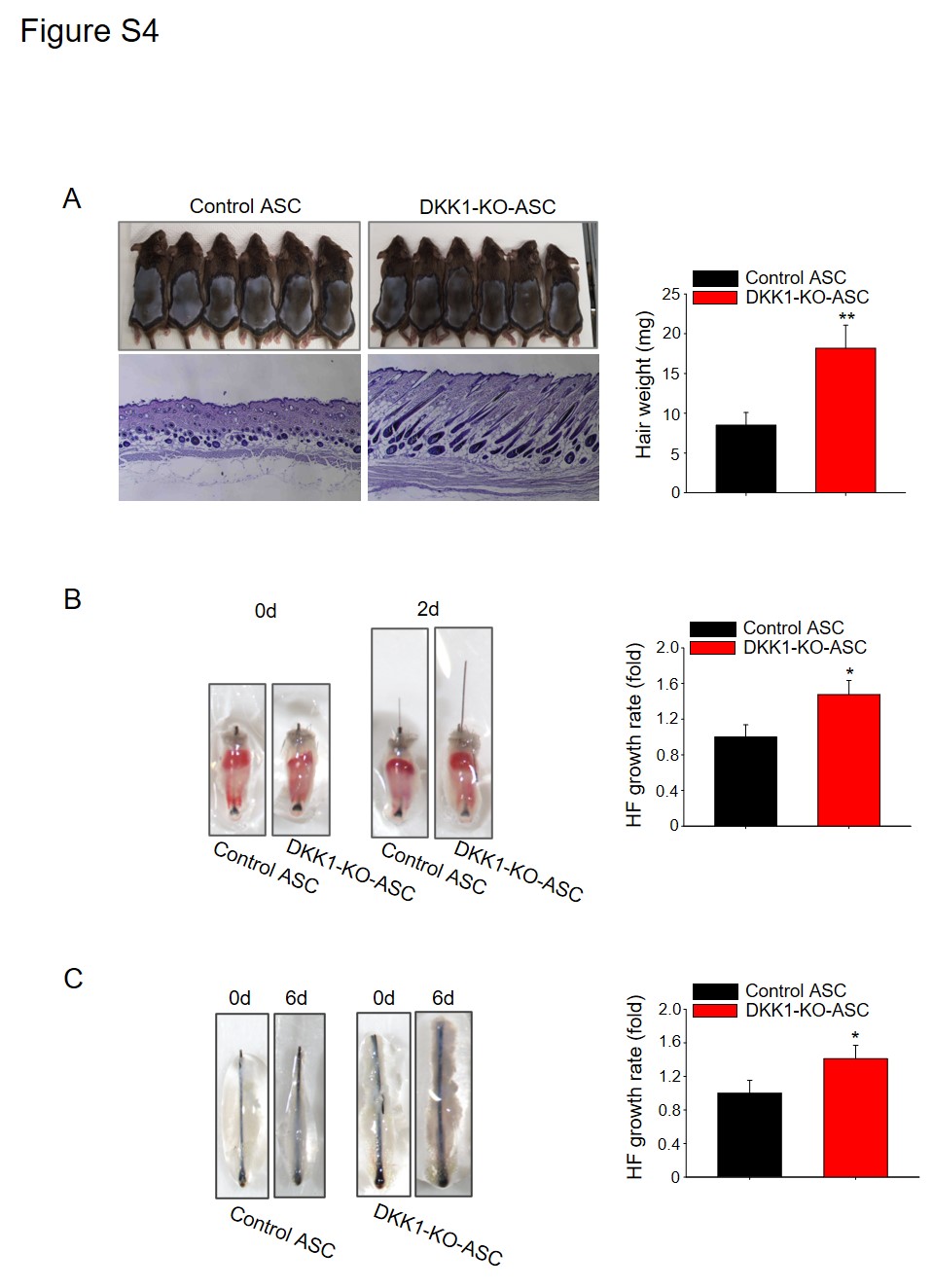

Supplement: Supplementary file 4 — Figure S4. Enhanced hair growth‐promoting effects of DKK1‐KO ASCs compared with naïve ASCs. (A) Injection of DKK1‐KO‐ASCs accelerated the transition from telogen to anagen in mice. (B, C) Treatment with conditioned medium obtained from DKK1‐KO‐ASCs significantly increased hair follicle (HF) growth in mouse (B) and human organ culture (C). More than 10 samples were analysed per group. *p < 0.05, **p < 0.01. [file CPR-57-e13562-s006.jpg]

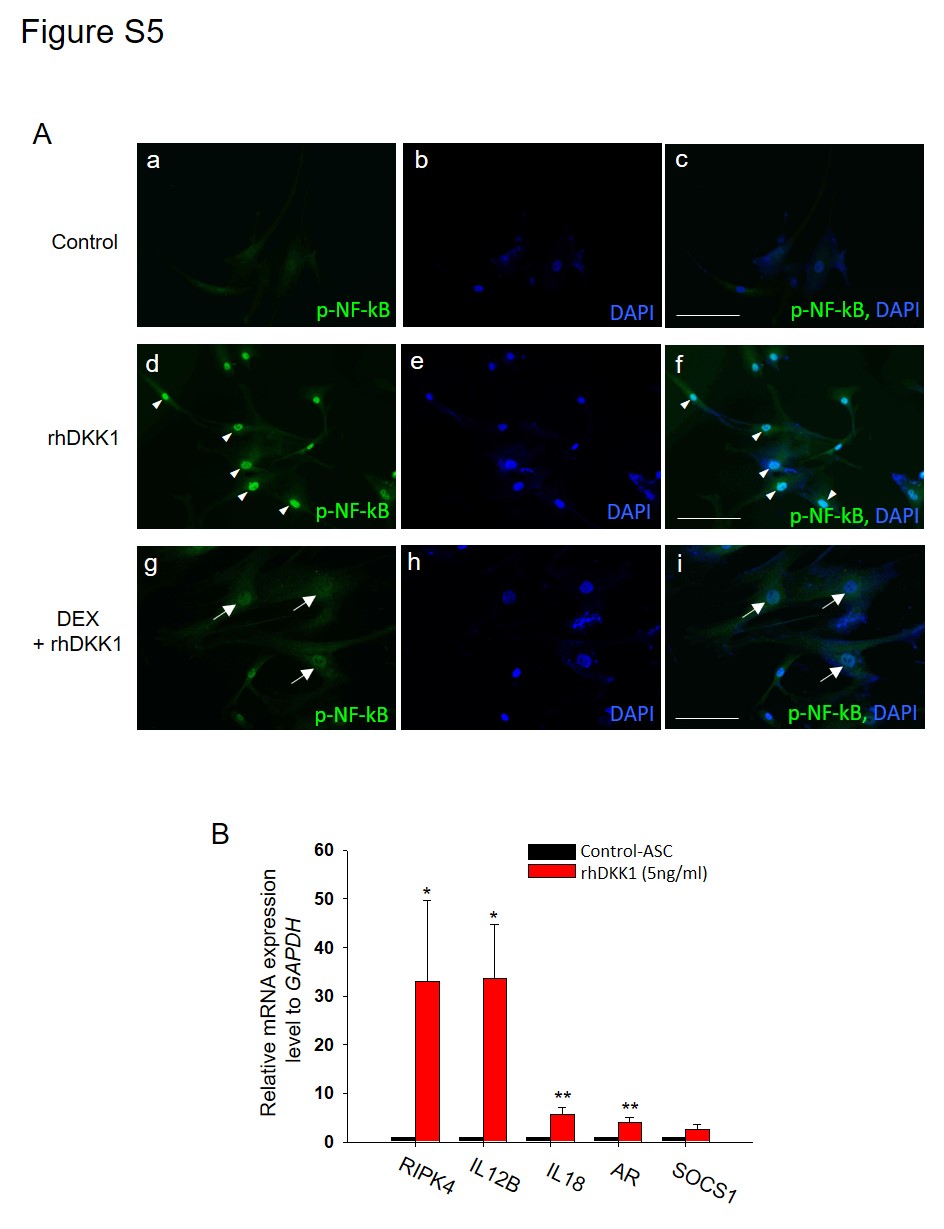

Supplement: Supplementary file 5 — Figure S5. Activation of the NF‐kB pathway by rhDKK1 treatment. (A) Naive ASCs were treated with rhDKK1 (5 ng/mL) for 30 min and were immunostained with phospho‐NF‐kB antibody. Prior to rhDKK1 treatment, the NF‐kB inhibitor (dexamethasone) was pre‐treated, and subsequent p‐NF‐kB immunostaining was performed. Control: A–C, rhDKK1 treatment; D–F, Dexamethasone and rhDKK1 treatment; G–I. Arrowheads indicate NF‐kB activation, while arrows NF‐kB inactivation. Scale bars indicate 10 μm. (B) The expression level of NF‐kB pathway‐related genes was assessed following rhDKK1 treatment in naïve ASCs. *p < 0.05, **p < 0.01. Three independent experiments were conducted for all QPCR data points. Error bars indicate the S.E.M. [file CPR-57-e13562-s001.jpg]

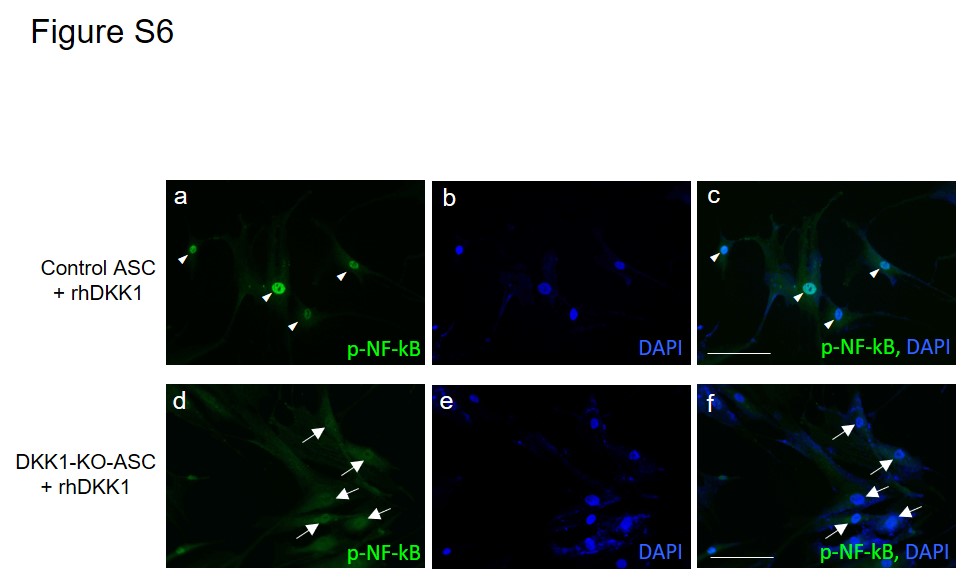

Supplement: Supplementary file 6 — Figure S6. Inactivation of NF‐kB in DKK1‐KO‐ASCs. Control‐ASCs and DKK1‐KO‐ASCs were subjected to treatment with rhDKK1 (5 ng/mL) for 30 min, followed by immunostaining with a phospho‐NF‐kB antibody. Arrowheads indicate NF‐kB activation, while arrows indicate NF‐kB inactivation. Scale bars indicate 10 μm. [file CPR-57-e13562-s002.jpg]

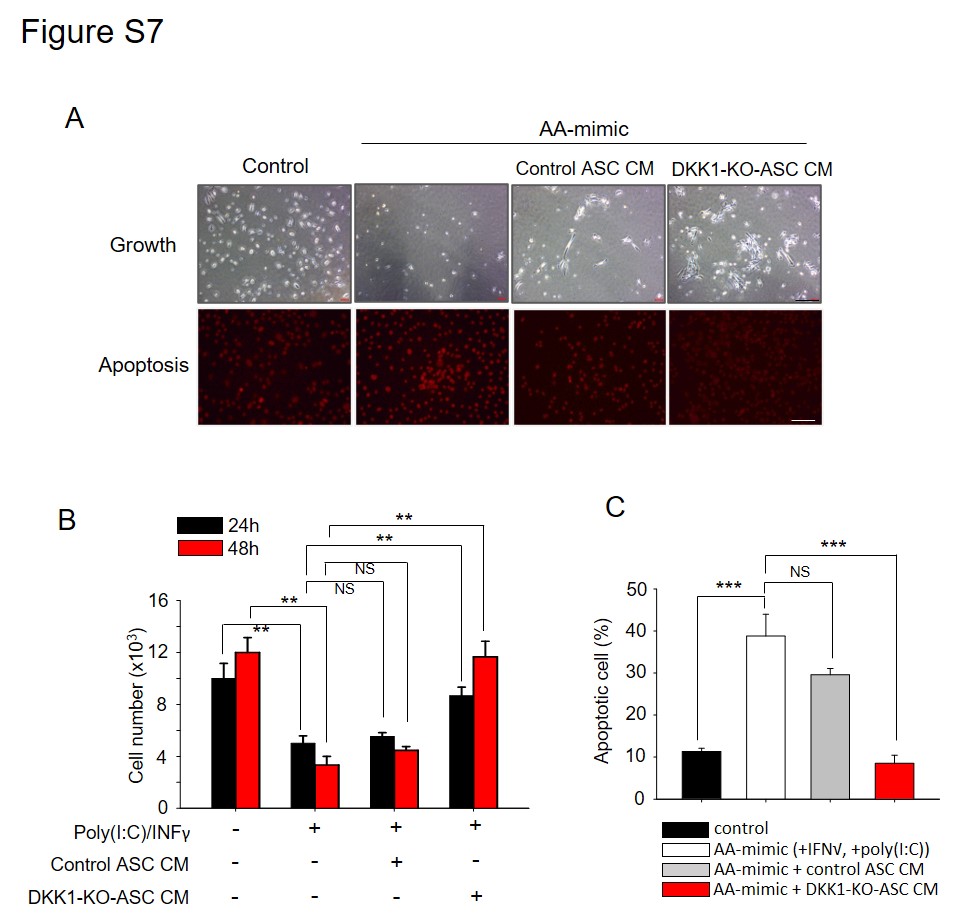

Supplement: Supplementary file 7 — Figure S7. Effect of DKK1‐KO‐ASCs on the AA‐mimicking cells. (A) Treatment with IFNγ and poly(I:C) induced cell death in ORS cells, and the impact of DKK1‐KO‐ASC treatment on cell death recovery was also examined. Scale bars indicate 10 μm. (B) ORS cell growth in response to IFNγ and poly(I:C) was assessed at 24 and 48 h. (C) ORS cell death was evaluated using apoptosis assay. **p < 0.01, ***p < 0.001. Three independent experiments were conducted for all data points. Error bars indicate the S.E.M. [file CPR-57-e13562-s008.jpg]
