# Supplementary material for: Involvement of DKK1 secreted from adipose‐derived stem cells in alopecia areata
Source: Cell Prolif. 2023 Nov 22;57(3):e13562. doi: 10.1111/cpr.13562 (PMC10905327; doi:10.1111/cpr.13562)
Supplement: Supplementary file 8 — Table S1. Primer list used for QPCR. [file CPR-57-e13562-s005.docx]

Table S1. Primer list used for QPCR

| Gene |  | Primer sequence |
| --- | --- | --- |
| DKK1 | Forward Primer | 5-CCTTGAACTCGGTTCTCAATTCC-3 |
|  | Reverse primer | 5-CAATGGTCTGGTACTTATTCCCG-3 |
| PKP1 | Forward Primer | 5-TTTGCCGTCGGACCAAAAGAT-3 |
|  | Reverse primer | 5-GAACCTCGATTGGAGTGGCTC-3 |
| FOXP3 | Forward Primer | 5-GTGGCCCGGATGTGAGAAG-3 |
|  | Reverse primer | 5-GGAGCCCTTGTCGGATGATG-3 |
| EDARDD | Forward Primer | 5-CCATTCAAGATACGGAACTCCC-3 |
|  | Reverse primer | 5-AGCAAGTCACTTATGGTGGGG-3 |
| RIPK4 | Forward Primer | 5-GATCTCCGGTTCCGAATCATC-3 |
|  | Reverse primer | 5-TCAGAAATCTTGACGTGGTAGTG-3 |
| ROR2 | Forward Primer | 5-TCCGAACGACCCTTTAGGAC-3 |
|  | Reverse primer | 5-TTTAGCCACCGCACGTTAGG-3 |
| KRT81 | Forward Primer | 5-GCTGAGAACGAGTTTGTGGCTC-3 |
|  | Reverse primer | 5-TCATACAGCCGCCTCAGGAAGT-3 |
| SOCS1 | Forward Primer | 5-CACGCACTTCCGCACATTC-3 |
|  | Reverse primer | 5-TAAGGGCGAAAAAGCAGTTCC-3 |
| IL18 | Forward Primer | 5-TCTTCATTGACCAAGGAAATCGG-3 |
|  | Reverse primer | 5-TCCGGGGTGCATTATCTCTAC-3 |
| IL12B | Forward Primer | 5-ACCCTGACCATCCAAGTCAAA-3 |
|  | Reverse primer | 5-TTGGCCTCGCATCTTAGAAAG-3 |
| RHEBL1 | Forward Primer | 5-TACCGCTGTGTAGGGAAGACA-3 |
|  | Reverse primer | 5-CCACTGTAGGATCGTAGCCTT-3 |
| AR | Forward Primer | 5-CCAGGGACCATGTTTTGCC-3 |
|  | Reverse primer | 5-CGAAGACGACAAGATGGACAA-3 |
